# Psychiatric morbidity and its recognition by doctors in patients with cancer

**DOI:** 10.1054/bjoc.2001.1724

**Published:** 2001-04

**Authors:** L Fallowfield, D Ratcliffe, V Jenkins, J Saul

**Affiliations:** CRC Psychosocial Oncology Group, School of Biological Sciences, University of Sussex, Falmer, BN1 9QG

**Keywords:** detection of psychiatric morbidity, communication

## Abstract

Psychiatric morbidity in patients with cancer is high and without appropriate treatment unremitting. We assessed the ability of 143 doctors to establish the psychological status of 2297 patients during outpatient consultations in 34 cancer centres and hospitals in the UK. Prior to seeing the doctor, consenting patients completed a short self-report questionnaire (GHQ12), designed for the psychological screening of large populations. At the end of the consultation, doctors completed visual analogue scales rating patients' distress. 837/2297 (36.4%) patients had GHQ scores suggestive of psychiatric morbidity. The doctors' sensitivity (true positive rate) was 28.87% (SD 25.29), specificity (true negative rate) 84.79% (SD 17.44). The misclassification rate was 34.7% (SD 13.79) meaning that for 797 patients the wrong assessment was probably made. These data show that much of the probable psychiatric morbidity experienced by patients with cancer goes unrecognized and therefore untreated. Doctors need communication skills training to elicit problems during consultations. Appropriate referrals to psychological services are necessary when patients requiring help are identified and ought to be an integral part of cancer care. © 2001 Cancer Research Campaign http://www.bjcancer.com


*British Journal of Cancer* (2001) **84**(8), 1011–1015
© 2001 Cancer Research Campaign
doi: 10.1054/ bjoc.2001.1724, available online at http://www.idealibrary.com on IDEAL®

http://www.bjcancer.com

### Psychiatric morbidity and its recognition by doctors in patients with cancer

**L Fallowfield, D Ratcliffe, V Jenkins and J Saul**

CRC Psychosocial Oncology Group, School of Biological Sciences, University of Sussex, Falmer BN1 9QG

**Summary** Psychiatric morbidity in patients with cancer is high and without appropriate treatment unremitting. We assessed the ability of 143 doctors to establish the psychological status of 2297 patients during outpatient consultations in 34 cancer centres and hospitals in the UK. Prior to seeing the doctor, consenting patients completed a short self-report questionnaire (GHQ12), designed for the psychological screening of large populations. At the end of the consultation, doctors completed visual analogue scales rating patients' distress. 837/2297 (36.4%) patients had GHQ scores suggestive of psychiatric morbidity. The doctors' sensitivity (true positive rate) was 28.87% (SD 25.29), specificity (true negative rate) 84.79% (SD 17.44). The misclassification rate was 34.7% (SD 13.79) meaning that for 797 patients the wrong assessment was probably made. These data show that much of the probable psychiatric morbidity experienced by patients with cancer goes unrecognized and therefore untreated. Doctors need communication skills training to elicit problems during consultations. Appropriate referrals to psychological services are necessary when patients requiring help are identified and ought to be an integral part of cancer care.
© 2001 Cancer Research Campaign http://www.bjcancer.com

**Keywords**: detection of psychiatric morbidity; communication

The stress associated with the diagnosis and treatment of cancer can cause significant psychiatric morbidity. Prevalence estimates vary depending on the assessment measures employed and the diagnostic criteria applied but reports for the past 3 decades (Maguire et al, 1978; Derogatis et al, 1983; Massie and Holland, 1990; Hopwood and Stephens, 2000) suggest that 25–50% of patients have psychological problems, with at least 25% meeting the criteria for either a major depressive disorder or adjustment disorder with depressed mood (Sellick and Crooks, 1999). These figures seem fairly consistent whether self-report questionnaires or standardized interviews are used and apply across all tumour sites and stages of disease, although there are some reports that the risk of major depression increases with recurrence (Cella et al, 1990; Hall et al, 1996) advanced disease, (Breitbart, 1995) and with increasing disability and pain (Passik et al, 1998). Evidence that any other patient or disease characteristics are associated with an increased likelihood of psychological morbidity is indeterminate.

Many of the psychological problems suffered by patients could be prevented or at least ameliorated if the communication skills of clinicians were better. In one study of women with breast cancer, those who were satisfied with the way in which the surgeon discussed their diagnosis and treatment options evidenced less psychiatric morbidity during the first year post treatment and at 2 and 3 year follow up (Fallowfield et al, 1990; Fallowfield and Hall, 1994). If psychological problems are recognized by health-care professionals then patients can be referred on for appropriate and effective interventions (Fallowfield, 1995). Given the extent of psychiatric morbidity and the proven efficacy of many different types of intervention for adult cancer patients (Meyer and Mark, 1995; Sheard and Maguire, 1999) this should form an important part of effective clinical management. Unfortunately several studies have shown that oncologists are not especially skilled at either discussing psychological problems in general, or at recognizing anxiety or depression (Hardman et al, 1989; Ford et al, 1994; Newell et al, 1998; Passik et al, 1998). Most published data suggest that doctors' ability to accurately detect psychological morbidity in patients is often little better than that due to chance.

Although the recognition of psychological morbidity is often hampered by the unwillingness of patients to disclose emotional concerns, the doctors are frequently reluctant to probe these areas satisfactorily (Hardman et al, 1989; Hopwood et al, 1991; Ford et al, 1996). For example in one study in which oncologists were tape-recorded giving patients bad news about their diagnoses and prognoses, the levels of psychological probing were low. When patients did volunteer psychological concerns, these were not pursued as oncologists kept the dialogue focused on a rigid biomedical agenda (Ford et al, 1996). Far too often psychological symptoms in patients are discounted as a normal consequence of having cancer (Fallowfield, 1988, 1990; Newell et al, 1998).

Even if doctors possess appropriate communication skills they often fail to utilize them given the stressful and difficult clinical environments in which many work. There are too few doctors seeing too many patients in UK cancer clinics with the added pressures of throughput and cost containment burdening them further. Furthermore surveys in the UK and US have shown that psychiatric morbidity and emotional burnout are almost as common in

*Received 1 August 2000*
*Revised 6 December 2000*
*Accepted 22 January 2001*

Lesley Fallowfield designed the study. All authors contributed to the collection and analysis of data and the writing of the report.

oncologists as in the patients whom they treat (Whippen and Canellos, 1991; Ramirez et al, 1995). Approximately 29% of British oncologists had above threshold scores (Ramirez et al, 1996) on a psychiatric screening inventory (GHQ12) (Goldberg and Williams, 1988). The under-trained, over-stretched and under-resourced doctor is unlikely to actively elicit information about the psychosocial impact that cancer and its treatment exerts on patients.

We report data from a study establishing the prevalence of probable psychiatric morbidity in a heterogeneous sample of 2297 patients with cancer seen in cancer clinics throughout the UK. We also report the ability of their doctors to detect this morbidity and the characteristics of both patient and doctor that were associated with detection.

### METHODS

143 doctors from 34 different cancer centres and hospitals in the UK took part in the study. All were participating in a randomized trial of a communication skills training programme for cancer specialists or specialists in training. Data about psychological morbidity and its recognition were collected as an outcome measure in part of the main trial. The specialty, seniority and sex of doctors are shown in Table 1. Participants included clinical, medical or surgical oncologists, 2 chest physicians who dealt primarily with lung cancer, a dermatologist with a specialist interest in melanoma and a palliative care physician.

2681 consecutive patients, waiting in out-patient clinics, were invited to join the main communication skills trial; of these 2331 (85%) gave their written consent to participate. The demographic characteristics of the 2297 patients with completed questionnaires for the detection of psychological morbidity study are shown in Table 2.

### Procedure

Prior to seeing the doctor consenting patients completed the 12 item version of the General Health Questionnaire (GHQ12). This instrument has high internal reliability and validity and was designed specifically as a brief screening instrument in large population studies (Goldberg and Williams, 1988). A conventional scoring method was employed with a threshold of 4 or more indicating probable caseness for psychological morbidity.

**Table 1** Characteristics of doctors (*n* = 143)

| Characteristics | *n* |
|---|---|
| Sex | |
| Male | 105 |
| Female | 38 |
| Previous communication skills training | |
| Yes | 6 |
| No | 137 |
| Seniority | |
| Junior (Specialist registrar) | 48 |
| Senior (Consultant) | 95 |
| Speciality | |
| Medical oncology | 54 |
| Clinical oncology | 60 |
| Surgical oncology | 25 |
| Dermatology, palliative care, chest medicine | 4 |

**Table 2** Characteristics of patients with GHQ ≥ 4

| | | GHQ ≥ 4 N (%) |
|---|---|---|
| Total sample | *n* = 2297 | 837 (36.4) |
| Age (years) | *n* (%) | |
| ≤ 30 | 112 (5.1) | 31 (27.7) |
| 31–50 | 572 (26.1) | 232 (40.6) |
| 51–70 | 1071 (48.9) | 393 (36.7) |
| >70 | 433 (19.8) | 147 (33.9) |
| Missing | 109 | |
| Sex | | |
| Male | 881 (38.4) | 300 (34.1) |
| Female | 1416 (61.6) | 537 (37.9) |
| Aim of treatment | | |
| Curative | 944 (41.1) | 299 (31.7) |
| Palliative | 849 (37.0) | 379 (44.6) |
| Remission | 215 (9.4) | 61 (28.4) |
| Uncertain | 289 (12.6) | 98 (33.9) |
| CA site | | |
| Breast | 662 (28.8) | 238 (36.0) |
| GI | 446 (19.4) | 158 (35.4) |
| Skin | 81 (3.6) | 22 (27.2) |
| Urological | 202 (8.8) | 69 (34.2) |
| CNS | 71 (3.1) | 36 (50.7) |
| Gynae | 153 (6.7) | 55 (35.9) |
| Head & Neck | 83 (3.6) | 33 (39.8) |
| Haematology | 196 (8.5) | 66 (33.7) |
| Lung | 172 (7.5) | 76 (44.2) |
| Musc/skeletal | 38 (1.7) | 14 (36.8) |
| Unknown 1⁰ | 33 (1.4) | 16 (48.5) |
| Other | 32 (1.4) | 13 (40.6) |
| Benign | 128 (5.6) | 41 (32.0) |

*All patients with valid GHQ (≤ 3 items omitted) and a doctor's rating for distress at end of consultation.

The doctors were blinded to the patients' GHQ scores but they were aware that the assessment was being made. Immediately following the patients' videotaped consultations, the doctors completed visual analogue scales (VAS) utilized in previous publications by ourselves and other authors in this type of study (Ford et al, 1994). On the VAS, the doctors self-rated various aspects of the interview including their perceptions of patients' psychological status at the beginning and end of the interview. The doctors' VAS ratings recorded for the patients at the end of their consultations were compared with each patient's GHQ score.

### RESULTS

### Statistics

Standard non-parametric and parametric statistical tests, $\chi^2$ and ANOVA were used throughout, with Bonferroni corrections for multiple comparisons applied as appropriate, using SPSS packages.

#### *Probable psychiatric morbidity*

Table 2 shows that 837/2297 (36.4%) of the patient group as a whole scored above threshold on the GHQ12 indicating probable psychiatric morbidity. Caseness was significantly higher in patients 31–50 years old ($\chi^2 = 9$, df = 3, $P = 0.029$) and in patients receiving palliative rather than curative treatment or those in remission ($\chi^2 = 40.75$, df = 3, $P = 0.0001$). The percentage of patients with high threshold scores within different cancer sites is

 © 2001 Cancer Research Campaign highest in those with CNS malignancies, unknown primaries and lung cancer. Of the patients seen by medical oncologists (includes 2 chest physicians, a dermatologist and palliative care specialist for ease of analysis), 332/948(35%) were probable cases, as were 385/983(39.2%) seen by clinical oncologists, and 120/366(32.8%) seen by surgeons. The number of probable cases seen by each specialist group differed significantly with clinical oncologists having more probable cases than the other two groups ($\chi^2 = 6.09$, df = 2, $P = 0.048$).

### *Doctors detection rates*

The mean number of consultations per doctor was 16 (SD 5, range 5–27) and each doctor saw on average 6 patients (SD 3, range 0–17) who scored above threshold. The more senior clinicians (consultants) saw a significantly greater number of patients with high GHQ scores than did the more junior specialist registrars ($\chi^2 = 5.56$, df = 1, $P = 0.018$). Specificity, sensitivity and misclassification rates are shown in Table 3. Overall the sensitivity or true positive rate of the doctors was 28.87%. Specificity or true negative rate was 84.79% and misclassification rate was 34.7%. These figures show that the doctors' predominant tendency was to assess patients as not distressed. Only 242 of the 827 patients with high scores were recognized as having probable psychiatric morbidity and therefore 595 patients with likely problems were missed. 1238 patients were correctly assessed as unlikely to have significant levels of distress to warrant a diagnosis of anxiety or depression but 222 patients with low GHQ scores were identified by the clinicians as probable cases of psychiatric morbidity. So overall a misclassification rate of 34.7% means that for 797 patients a potentially incorrect assessment was made.

There was no significant difference in sensitivity and specificity rates according to specialty but there was a difference in misclassification rates (F = 3.15, df = 2, $P = 0.046$); the clinical oncologists who saw more high GHQ scorers, also had a significantly higher misclassification rate than did surgeons and medical oncologists (Bonferroni test, $P < 0.05$). When seniority was taken into consideration, there was no effect of specialty amongst more junior doctors; the difference remained only for the senior clinical oncologists who misclassified a significantly greater number of patients as distressed (Bonferroni test, $P < 0.05$).

When data were scrutinized according to whether or not the patient had been seen by the doctor on a previous occasion there was no effect on detection rates. There were no differences in detection rates according to the sex of the doctor.

### Length of consultations

The mean length of consultations was 13.49 minutes, (mode 10 min, range 1–67 min). There was a significant difference in mean times between groups (F = 42.15, df = 3, $P = 0.0000$) with the surgeons having the shortest consultation times (Bonferroni $P < 0.05$). Overall there was a significant difference in the length of consultation and the detection rates of probable psychiatric morbidity (F = 52.93, df = 3, $P = 0.0000$). Patients who were true positives had longer mean consultations than did those who were false negatives (17.68 mins v 14.94 mins, t = 4.08, df = 818, $P = 0.0000$, SE = 0.672, CI: 1.42–4.05).

### DISCUSSION

These results from a very large heterogeneous sample of patients with cancer reinforce previous findings from earlier, smaller studies that psychological morbidity is still common and that much of it goes unrecognised and is not therefore treated. The doctors were all aware that the psychological status of their patients was being assessed and that their interactions were being videotaped. Therefore it is likely that the situation when doctors are not being monitored is even bleaker. Patients with problems that could potentially be ameliorated are not getting the psychological support required. Many clinicians would claim that they leave this area for their specialist nurses to pick up on, but there is little evidence that such nurses screen effectively for psychological morbidity either (Heaven and Maguire, 1997). Furthermore, many nurses are just as burdened as the doctors by the volume of patients and time pressures, so many patients with psychological problems get missed. Time did appear to be a relevant factor as true positive patients i.e. those who were correctly identified by the doctor, had significantly longer consultations than those who were false negatives, that is patients with probable psychological morbidity who were missed by the doctors. Until an associated project looking at utterance by utterance analysis of the videotaped interactions is completed, we will not know if the increased length of time was due to the doctors eliciting the psychological problems and exploring them effectively in the recognized cases; alternatively patients who were missed might have given clear cues during the consultation which the doctor failed to acknowledge, or maybe chose not to follow-up. It has been shown in a general practise setting that doctors who recognise depression communicate rather differently from those who do not, in particular they ask more open questions about feelings and affect (Carney et al, 1999). We also

**Table 3** Specificity, sensitivity and misclassification rates by sex, seniority and specialty of clinician

| | | Specificity | | | Sensitivity | | | Misclassification | | |
|---|---|---|---|---|---|---|---|---|---|---|
| | *n* | % | SD | CI | % | SD | CI | % | SD | CI |
| Total sample | 143 | 84.79 | 17.44 | 81.91–87.67 | 28.87 | 25.29 | 24.69–33.05 | 34.70 | 13.79 | 32.42–36.98 |
| Male | 105 | 84.51 | 16.44 | 81.32–87.69 | 29.58 | 24.62 | 24.82–34.35 | 34.69 | 12.83 | 32.21–37.17 |
| Female | 38 | 85.59 | 20.18 | 78.96–92.22 | 26.91 | 27.31 | 17.94–35.89 | 34.74 | 16.35 | 29.37–40.12 |
| Sp Registrar | 48 | 87.41 | 16.31 | 82.68–92.15 | 26.85 | 27.81 | 18.78–34.93 | 32.23 | 12.72 | 28.54–35.93 |
| Consultant | 95 | 83.47 | 17.92 | 79.82–87.12 | 29.89 | 24.00 | 25.00–34.79 | 35.95 | 14.21 | 33.06–38.85 |
| Med Oncologist | 58 | 80.85 | 19.08 | 75.83–85.87 | 33.09 | 23.84 | 26.82–39.36 | 34.00 | 15.32 | 29.97–38.03 |
| Clinical Oncologist | 60 | 86.51 | 17.02 | 82.12–90.91 | 25.31 | 26.57 | 18.44–32.17 | 37.52 | 13.06 | 34.15–40.89 |
| Surgeon | 25 | 89.82 | 12.32 | 84.73–94.90 | 27.65 | 24.95 | 17.35–37.95 | 29.57 | 10.05 | 25.43–33.72 |

These were calculated by taking the mean score for each doctor and then the mean for each variable know from a previous study of 178 senior doctors in cancer medicine attending communication skills courses that eliciting and then dealing effectively with patients' emotional problems caused them considerable difficulty (Fallowfield et al, 1998).

There are at least 2 potential problems with the interpretation of our results that need acknowledgement. First any screening instrument by definition, will have imperfect sensitivity and specificity itself, although there have been at least 6 studies showing that both of these are very good for the GHQ. There is no true gold standard against which to assess the clinician's ability to detect psychiatric morbidity, but (Goldberg et al, 1993) provide an example of how the GHQ12 has been used successfully in studies aimed at helping general practitioners to identify distress in their patients. Similarly the GHQ28 was used to evaluate physicians' skills in reducing patients' distress in an RCT of a training intervention in the US (Roter et al, 1995). Nevertheless in our study at least some of the clinicians' false-negative patients, could in fact have been rated following an accurate clinical appraisal of transient distress and dysfunction; such patients might have an above threshold score on the GHQ but might not have a psychiatric illness following a standardized psychiatric interview. We also recognize that the content of the consultation may have reduced or increased the patients' overt signs of distress making doctors appear less accurate. We do not have any way of knowing what frame of reference or what criteria the doctors used to determine distress in their patients when they completed the visual analogue scales. Nevertheless we do know from previous work that doctors tend to exhibit very low levels of psychosocial probing during consultations (Ford et al, 1996). There is an increasing body of evidence showing that education programmes for clinicians can improve their recognition and management of depression (Roter et al, 1995; Stewart, 1996; Gerrity et al, 1999) and that the general communication skills of healthcare professionals working within oncology are enhanced following well structured training courses (Fallowfield et al, 1998; Baile et al, 1999; Maguire, 1999).

The magnitude of the problem identified by this study cannot be overestimated. Cancer incidence in the UK is more than 250 000 per annum and approximately one-third of these patients are likely to have psychological disorders that merit intervention. The prevalence of anxiety and depression following the diagnosis and treatment of cancer does diminish slightly over time but it never returns to levels seen in the general population even if the treatment has been curative. Not all patients require the help of a psychiatrist or psychologist, nor are the resources for these supportive services always available within oncology clinics, so much of the psychological morbidity has to be managed by oncologists and specialist nurses. Screening patients using validated questionnaires prior to seeing the doctor might help improve detection of potential psychological morbidity and some interesting data have been published showing the acceptability and utility of automated (touchscreens) screening devices in busy oncology clinics (Velikova et al, 1999). Patients with high scores still require proper clinical assessment; if the clinician lacks either the necessary interviewing skills, or the inclination to probe further, they can at least refer distressed patients on for more specialist help on an informed basis. Furthermore there is evidence for improved efficacy of psychological interventions in screened populations (Sheard and Maguire, 1999).

If the manifest psychological distress experienced by patients with cancer is to be prevented and/or ameliorated then urgent attention must be given to helping doctors to improve their communication skills in general and with their recognition of potentially treatable anxiety and depression.

### ACKNOWLEDGEMENTS

The authors wish to acknowledge the help of Anthony Duffy, Anna Fair, Caroline Fallowfield, Sarah Ford, Angela Hall, Philippa Jones, and Anna Souhami with data collection. We are also very grateful to the patients and doctors who took part in the study and for the help and co-operation of clinic staff. The study was funded by the Cancer Research Campaign.

### REFERENCES

Baile W, Kudelka AP, Beale EA, Glober GA, Myers MA, Greisinger AJ, Bast RC, Goldstein MG, Novack D and Lenzi R (1999) Communication skills training in oncology. *Cancer* **86**: 887–897

Breitbart W (1995) Identifying patients at risk for, and treatment of major psychiatric complications of cancer. *Support Care Cancer* **3**: 45–60

Carney PA, Eliassen MS, Wolford GL, Owen M, Badger LW and Dietrich AJ (1999) How physician communication influences recognition of depression in primary care. *J Fam Pract* **48**: 958–964

Cella DF, Mahon SM and Donovan MI (1990) Cancer recurrence as a traumatic event. *Behav Med* **16**: 15–22

Derogatis LR, Morrow GR, Fetting J, Penman D, Piasetsky S, Schmale AM, Henrichs M and Carnicke CL, Jr (1983) The prevalence of psychiatric disorders among cancer patients. *JAMA* **249**: 751–757

Fallowfield LJ (1988) The psychological complications of malignant disease. In *Medical Complications of Malignant Disease*, Kaye, SB and Rankin EM (eds), Vol. 2 (2) July 1988. pp. 461–478. Bailliere's Clinical Oncology: International Practice and Research. Balliere Tindall: London.

Fallowfield LJ (1990) *The Quality of Life: The Missing Measurement in Health Care*. Souvenir Press: London.

Fallowfield LJ (1995) Psychosocial interventions in cancer. *BMJ* **311**: 1316–1317

Fallowfield LJ and Hall A (1994) Psychological effects of being offered choice of surgery for breast cancer. *BMJ* **309**: 448

Fallowfield LJ, Hall A, Maguire GP and Baum M (1990) Psychological outcomes of different treatment policies in women with early breast cancer outside a clinical trial. *B M J* **301**: 575–580

Fallowfield LJ, Lipkin M and Hall A (1998) Teaching senior oncologists communication skills: results from phase 1 of a comprehensive longitudinal program in the UK. *J Clin Oncol* **16**: 1961–1968

Ford S, Fallowfield LJ and Lewis S (1994) Can oncologists detect distress in their out-patients and how satisfied are they with their performance during bad news consultations? *B J Cancer* **70**: 767–770

Ford S, Fallowfield LJ and Lewis S (1996) Doctor-patient interactions in oncology. *So Sci Med* **42**: 1511–1519

Gerrity MS, Cole SA, Dietrich AJ and Barrett JE (1999) Improving the recognition and management of depression: is there a role for physician education? *J Fam Pract* **48**: 949–957

Goldberg D and Williams P (1988) *A User's Guide to the General Health Questionnaire* NFER-Nelson: Windsor

Goldberg DP, Jenkins L, Millar T and Faragher EB (1993) The ability of trainee general practitioners to identify psychological distress among their patients. *Psychol Med* **23**: 185–193

Hall A, Fallowfield LJ and A'Hern RP (1996) When breast cancer recurs: a 3-year prospective study of psychological morbidity. *The Breast J* **2**: 197–203

Hardman A, Maguire P and Crowther D (1989) The recognition of psychiatric morbidity on a medical oncology ward. *J Psychosom Res* **33**: 235–239

Heaven CM and Maguire P (1997) Disclosure of concerns by hospice patients and their identification by nurses. *Palliat Med* **11**: 283–290

Hopwood P and Stephens RJ (2000) Depression in patients with lung cancer: prevalence and risk factors derived from quality-of- life data [In Process Citation]. *J Clin Oncol* **18**: 893

Hopwood P, Howell A and Maguire P (1991) Screening for psychiatric morbidity in patients with advanced breast cancer: validation of two self-report questionnaires. *B J Cancer* **64**: 353–356

 *© 2001 Cancer Research Campaign*

Maguire GP, Lee EG, Bevington DJ, Kuchemann CS, Crabtree RJ and Cornell CE (1978) Psychiatric problems in the first year after mastectomy. *BMJ* **1**: 963–965

Maguire P (1999) Improving communication with cancer patients. *Eur J Cancer* **35**: 2058–2065

Massie MJ and Holland JC (1990) Depression and the cancer patient. *J Clin Psychiatry* **51 Suppl**: 12–7; discussion 18–19

Meyer TJ and Mark MM (1995) Effects of psychosocial interventions with adult cancer patient: a meta analysis of randomised experiments. *Health Psycho* **14**: 101–108

Newell S, SansonFisher RW, Girgis A and Bonaventura A (1998) How well do medical oncologists' perceptions reflect their patients' reported physical and psychosocial problems? – Data from a survey of five oncologists. *Cancer* **83**: 1640–1651

Passik SD, Dugan W, McDonald MV, Rosenfeld B, Theobald DE and Edgerton S (1998) Oncologists' recognition of depression in their patients with cancer. *J Clin Oncol* **16**: 1594–1600

Ramirez AJ, Graham J, Richards MA, Cull A, Gregory WM, Leaning MS, Snashall DC and Timothy AR (1995) Burnout and psychiatric disorder among cancer clinicians. *Br J Cancer* **71**: 1263–1269

Ramirez AJ, Graham J, Richards MA, Cull A and Gregory WM (1996) Mental health of hospital consultants: the effects of stress and satisfaction at work. *The Lancet* **347**: 724–728

Roter DL, Hall JA, Kern DE, Barker LR, Cole KA and Roca RP (1995) Improving physicians' interviewing skills and reducing patients' emotional distress. A randomized clinical trial. *Arch Intern Med* **155**: 1877–1884

Sellick SM and Crooks DL (1999) Depression and cancer: an appraisal of the literature for prevalence, detection, and practice guideline development for psychological interventions. *Psychooncology* **8**: 315–333

Sheard T and Maguire P (1999) The effect of psychological interventions on anxiety and depression in cancer patients: results of two meta-analyses. *Br J Cancer* **80**: 1770–1780

Stewart MA (1996) Effective physician-patient communication and health outcomes: a review. *Can Med Assoc J* **152**: 1423–1433

Velikova G, Wright EP, Smith AB, Cull A, Gould A, Forman D, Perren T, Stead M, Brown J and Selby PJ (1999) Automated collection of quality-of-life data: a comparison of paper and computer touch-screen questionnaires. *J Clin Oncol* **17**: 998–1007

Whippen DA and Canellos GP (1991) Burnout syndrome in the practice of oncology: results of a random survey of 1000 oncologists. *J Clin Oncol* **9**: 1916–1920

*© 2001 Cancer Research Campaign*
